# PrEP Familiarity, Interest, and Usage Among 364 Black and Hispanic Adults in Indiana

**DOI:** 10.3389/fpubh.2022.810042

**Published:** 2022-05-06

**Authors:** Jessica T. Campbell, Olivia R. Adams, Margaret Bennett-Brown, Brennan Woodward, Amanda N. Gesselman, Gregory Carter

**Affiliations:** ^1^The Kinsey Institute, Indiana University, Bloomington, IN, United States; ^2^Department of Gender Studies, Indiana University, Bloomington, IN, United States; ^3^Department of Nursing, Indiana University, Bloomington, IN, United States; ^4^Department of Communication Studies, Texas Tech University, Lubbock, TX, United States

**Keywords:** HIV pre-exposure prophylaxis (PrEP), PrEP, PrEP usage, PrEP interest, PrEP familiarity

## Abstract

Pre-exposure prophylaxis, or PrEP, is a once-daily preventative prescription pill against HIV for adults or adolescents who have sex or inject drugs. PrEP may be especially useful among Black and Hispanic Americans, who are particularly at risk for HIV in the United States. In spite of this vulnerability, rates of PrEP use in Black and Hispanic communities are low. Here, we examined familiarity with, prior usage of, and future interest in PrEP among 364 Black and Hispanic Indiana residents. Indiana is an important context for this work, due to severe HIV outbreaks in the area over the last 8 years. Around half of all participants had never heard of PrEP, with Hispanic participants being less familiar than Black participants. Prior PrEP use was low, at around 10%, and was lower for Hispanic than Black participants. Around 21% of all participants reported interest in PrEP after learning of it in our study. Further, participants identified strategies that would make discussions about PrEP with a medical provider more comfortable. Black and Hispanic participants reported feeling the most comfortable with addressing PrEP usage with providers if: (a) the provider was the one who brought up the subject of PrEP, (b) there was written information available to the patient (i.e., brochures), and (c) the patient already knew they qualified for the prescription in terms of personal eligibility and insurance coverage. Additional provider and patient education, as well as openness on the part of the provider, can help to lessen the disparities associated with PrEP need and actual PrEP usage.

## Introduction

Pre-exposure prophylaxis, or PrEP, is a once-daily preventative prescription pill against HIV for adults or adolescents who have sex or use injection drugs (1, 2). The term “PrEP” has most often referred to Truvada^®^ (tenofovir disoproxil fumarate [TDF]/emtricitabine) but now encompasses the newer version that is now available under the brand name Descovy^®^ (tenofovir alafenamide [TAF]/emtricitabine); however, Descovy^®^ has not been tested on people who have receptive vaginal sex or inject drugs, so it is not yet accessible for all populations (3). As next-generation PrEP is coming forward, such as the injectable cabotegravir, the term will increasingly refer to a general group of medications that prevent HIV infection (4).

Studies have shown that PrEP helps lower the chances of getting HIV from sex by 99% and from intravenous drug use by ~74% when taken daily (5). PrEP is thus a valuable tool for addressing HIV and reducing national rates of new infections. Although PrEP usage is becoming more common in the United States, clear disparities remain with regards to at-risk demographic subgroups (6, 7) and geographical location outside of urban areas (8, 9). In the current study, we investigated familiarity, prior usage, and current interest in PrEP in a sample of Black and Hispanic adults in the state of Indiana. We also gathered information on strategies for increasing Black and Hispanic participants' comfort with discussing PrEP in a healthcare setting.

Black and Hispanic people are among the most at risk for HIV in the U.S. Black Americans in particular account for 13% of the U.S. population but accounted for 42% of HIV infections in 2019 (10). Hispanic/Latinx Americans comprise 18% of the U.S. population (10), but accounted for 30% of HIV infections in 2019 (10). According to data collected between 2015 and 2019, Black and Hispanic/Latino men had the highest number of HIV diagnoses per 10,000 cases (11). Further, the rate of diagnoses stayed stable for these racial/ethnic groups over time, while the rate of diagnoses for white men decreased by 17% (11). Women are also at substantially greater risk: Black women are over 14 times more likely to die from HIV compared to white women, and Hispanic/Latinx women die from HIV at three times the rate of white women (10).

The American Midwest has historically experienced the lowest HIV rates by region (12). However, in 2014, Indiana's Scott County became the site of the largest HIV outbreak related to intravenous drug use, leading the state's Governor to declare a public health emergency (13). In 2021, counties in six Midwest states—Illinois, Indiana, Michigan, and Ohio—were identified by the U.S. Department of Health and Human Services as priority jurisdictions for addressing the transmission of HIV (5). These and other proximal areas had already been identified as vulnerable for the rapid dissemination of HIV in years prior (14).

Racial disparities in Midwest HIV diagnoses abound: recent estimates show that around half of all new HIV diagnoses were reported among Black people, and 13% among Hispanic people (15). In Indiana, estimates from 2018 indicate that the number of Black individuals living with HIV were nearly seven times the number of white individuals living with HIV; there were nearly three times the amount of Hispanic/Latinx (vs. white) individuals living with HIV (16). Additionally, Black individuals make up 44 per 100,000 new HIV diagnoses in Indiana; Hispanic/Latinx individuals make up 16, and white individuals make up 5 per 100,000 (16).

Although they are among the most at risk for HIV, there are lower rates of PrEP usage among Black people and Hispanic people (17, 18). For example, in 2015 and 2016, fewer than 15,000 PrEP prescriptions were filled for Black and Hispanic people in the U.S., though research suggests that PrEP may have been useful for as many as 500,000 Black and 300,000 Hispanic people at that time (15). In addition to barriers like financial burden and social stigma, the lack of awareness or familiarity with PrEP is a substantial hindrance to access. Data from the National HIV Behavioral Surveillance project has shown that Black and Hispanic men who have sex with men (MSM) were less likely to have heard about PrEP than were white MSM (19). Similarly, Hispanic people are also less likely than their white counterparts to be aware of PrEP with only about 25% of individuals being aware of the medication upon testing for HIV (20). Existing research has indicated that language and cultural barriers may inhibit the familiarity with, and subsequent access to, PrEP as well as HIV testing and treatment (21). Generally speaking, studies conducted in urban settings have shown a reduction in this disparity, with between 86 and 95% of MSM in all racial/ethnic categories having familiarity with PrEP (22). PrEP familiarity in non-urban areas remains low (23).

Studies focusing on women and PrEP also suggest significant disparities in knowledge, intervention strategies, and research efforts despite the high levels of HIV transmission and deaths within this population, particularly among Black and Hispanic women (24). While many HIV prevention campaigns and studies focus on the MSM and intravenous drug use populations, heterosexual contact accounted for 92% of HIV infections among Black women and 68% for White women in 2015 (25). Furthermore, in a review of studies on interventions to increase PrEP uptake, only one study in the existing literature focused on cisgender women (26).

Among studies examining cisgender and heterosexual women's knowledge of PrEP, between 0 and 33% of women had ever heard of the preventative drug across studies from 2000 to 2018 (27). In a more recent community survey of 53 low income Black women living in an urban setting, Hill et al. (28) report that 37% of participants had heard of PrEP, but only 16% knew that it was specifically used for HIV prevention. Shende et al. (29) suggest a similarly low level of PrEP knowledge among Hispanic women—in their study, 88% reported never having learned about PrEP before participating. Despite these low levels of PrEP knowledge, women frequently express interest in it after learning more about its role in HIV prevention. For Black women, PrEP offers special benefits as it circumvents the need for partner negotiation that other HIV prevention methods like condom use require (28, 30).

Importantly, when informed about PrEP, participants generally express positive attitudes toward and interest in pursuing a PrEP prescription (8, 30). While much of the existing literature has focused on potential barriers to PrEP (31–33), relatively few have explored patient preferences for requesting PrEP in a medical setting. Sewell et al. (34) found that patient-led decision making about PrEP usage is supportive of patient autonomy and that universal offering of PrEP can support uptake of the medication among a broader range of populations. However, Sewell et al. (34) also noted that a lack of both patient and provider knowledge requires intentional education and preparation to facilitate PrEP centered conversations. In addition to our assessment of familiarity, prior usage, and interest in future use of PrEP, we also asked participants to identify strategies that could be enacted in the healthcare setting to make them more comfortable with asking for or discussing PrEP. Broadening the positive impact of PrEP requires accessibility, availability, and comfortable/safe dynamics for education of the medication (35). Recognizing gaps in knowledge of PrEP is crucial for informing future targeted interventions and adding more inclusive perspectives to the literature (36). We assessed Black and Hispanic people living in Indiana.

## Study Overview

The present research seeks to identify familiarity with, interest, and usage of PrEP among Black and Hispanic participants in Indiana to better understand how to serve wider demographic groups with PrEP. Specifically, we examine four research questions:

1. Do Black and Hispanic people differ in their knowledge of PrEP?
2. Do Black and Hispanic people differ in their interest in PrEP?
3. Do Black and Hispanic people differ in their usage of PrEP?
4. What are the reported preferences for asking about PrEP in a medical setting?

## Methods

### Data Collection

Data were collected from March 19th, 2021, to April 16th, 2021. These data were part of a larger study on the multifaceted healthcare experiences of people of color residing in the state of Indiana. Participants were recruited from Qualtrics Panels, a market research panel operated by the survey software company Qualtrics (www.qualtrics.com). These Panels are composed of participants who have agreed to take part in online surveys. Inclusion criteria were that the participant must reside in the state of Indiana, identify as a person of color, and be 18 years of age or older.

### Participants

To be included in the analysis, participants had to report (1) they were at least 18 years old, (2) they identified most strongly with being Black or Hispanic, and (3) they live in the state of Indiana. Of the 364 participants, 275 identified as Black and 89 identified as Hispanic. Most participants were heterosexual, single, and female. See Table 1 for complete participant demographics.

**Table 1:** Participant demographics.

	**Black participants (*n* = 275)**	**Hispanic participants (*n* = 89)**
**Age group (** ***n)***		
18–24 years old	54.2% (149)	42.9% (27)
25–34 years old	17.5% (48)	20.6% (13)
35–44 years old	11.6% (32)	28.6% (18)
45–54 years old	9.1% (25)	1.6% (1)
55–64 years old	4.7 % (13)	4.8% (3)
65–74 years old	2.5% (7)	1.6% (1)
75 years old or older	0.4% (1)	0.0% (0)
**Gender identity (** ***n*** **)**		
Male	29.1% (80)	41.6% (37)
Female	67.6% (186)	55.1% (49)
Transgender	1.5% (4)	0.0 % (0)
Non-binary	1.8% (5)	3.4% (3)
**Sexual orientation (** ***n*** **)**		
Heterosexual	77.5% (213)	66.3% (59)
Gay	1.8% (5)	2.2% (2)
Lesbian	5.1% (14)	3.4% (3)
Bisexual	12.4% (34)	20.2% (18)
Other	3.3% (9)	7.8% (7)
**Education (** ***n*** **)**		
Less than a high school degree	5.1% (14)	9.0% (8)
High school degree / GED	36.0% (99)	28.1% (25)
Some college but no degree	23.6% (65)	22.5% (20)
Associate degree	13.5% (37)	12.4% (11)
Bachelor's degree	11.6% (32)	19.1% (17)
Master's degree	5.1% (14)	6.7% (6)
Doctoral or professional degree	5.1% (14)	2.2% (2)
**Current relationship status (** ***n*** **)**		
Single	57.5% (158)	34.9% (22)
In a committed relationship	20.4% (56)	28.6% (18)
Married	14.9% (41)	27.0% (17)
Separated	2.2% (6)	4.8% (3)
Divorced	3.3% (9)	3.2% (2)
Widowed	1.8% (5)	1.6% (1)

### Measures

#### Sociodemographics

Participants reported their age group, gender (male; female; transgender; non-binary), sexual orientation (heterosexual, gay, lesbian, bisexual, or other), level of education, and relationship status (single; in a committed relationship; married; separated; divorced; widowed).

#### Knowledge of PrEP

Participants were given a brief description of PrEP and then responded to the question “Before today have you heard of PrEP?” where 1 = *Yes*, 2 = *Yes, but I didn't know what for*, and 3 = *No*.

#### Interest in PrEP

Participants responded to the question, “Are you interested in getting PrEP for yourself?” where 1 = *Definitely yes*, 2 = *Probably yes*, 3 = *Not sure*, 4 = *Probably not*, 5 = *Definitely not*.

#### PrEP Usage

Participants responded to the question, “Are you currently on PrEP?” where 1 = *Yes*, 2 = *No*, and 3 = *No, but I have been in the past*.

#### Making PrEP Conversations More Comfortable With Providers

Participants were asked a series of questions that were prefaced with, “Which of these would make you feel more comfortable asking a doctor for PrEP?” Participants responded with a “1” if they believed it made PrEP discussions more comfortable for them. Participants were told to select all options that they agreed with. Options included providing brochures in the lobby, and having a provider bring up PrEP. A complete list of options and responses are available in **Table 5**.

## Results

### Data Analysis Plan

Below, we report frequencies for the overall sample and by racial/ethnic subgroup. We report bivariate correlations, showing the association between racial groups and the three variables of interest, as well as how those variables of interest relate to one another. Next, we present independent samples *t*-tests to assess race/ethnicity-related differences in knowledge of PrEP, interest in PrEP, and PrEP usage. Finally, we report frequencies regarding preferred methods for asking about PrEP in a medical setting.

### Frequencies of PrEP Knowledge, Usage, and Interest for Overall Sample and Racial/Ethnic Subgroups

In the overall sample, 30.5% had heard of PrEP before and 11.6% had used PrEP before. An additional 20.9% had heard of PrEP but did not know what the medication was used for. After learning about PrEP in the study, 23% reported that they were “definitely” or “probably” interested in getting PrEP for themselves.

For Black participants, 55.3% had heard of PrEP before, though 22.9% did not know what the medication was used for. 12.4% of Black participants had used PrEP before. After learning about PrEP in the study, 23.3% reported that they were “definitely” or “probably” interested in getting PrEP for themselves. An additional 34% of Black participants were unsure of their personal interest in PrEP.

For Hispanic participants, 39.3% had heard of PrEP before, though 14.6% did not know what the medication was used for. Nine percent of Hispanic participants had used PrEP before. After learning about PrEP in the study, 21.8% reported that they were “definitely” or “probably” interested in getting PrEP for themselves. An additional 32% of Hispanic participants were unsure of their personal interest in PrEP.

### Bivariate Correlations Between Race/Ethnicity With PrEP Knowledge, Usage, and Interest

In the overall sample, knowledge of PrEP was positively related to PrEP usage (*r* = 0.22, *p* < 0.001). Interestingly, knowledge of PrEP was negatively related to endorsing the notion that people should take PrEP (*r* = −0.18, *p* < 0.001). See Table 2 for complete correlations.

**Table 2:** Correlations matrix.

**Variable**	**1**	**2**	**3**
**Overall sample**			
1. Knowledge of PrEP^a^	—		
2. Interest in PrEP^b^	−0.023	—	
3. Currently on PrEP^c^	0.215^***^	−0.067	—
**Black participants**			
1. Knowledge of PrEP	—		
2. Interest in PrEP	−0.049	—	
3. Currently on PrEP	0.219^***^	−0.043	—
**Hispanic participants**			
1. Knowledge of PrEP	—		
2. Interest in PrEP	0.048	—	
3. Currently on PrEP	0.159	−0.124	—

*** *p < 0.001 (two-tailed)*.

a *Higher scores represent less familiarity with PrEP*.

b *Higher scores represent a lower interest in getting PrEP*.

c *Higher values indicate not being on PrEP*.

Among Black participants, knowledge of PrEP was positively related to PrEP usage (*r* = 0.22, *p* < 0.001). Among Hispanic participants, there were no significant correlations among PrEP knowledge, usage, and interest; see Table 2 for complete correlations.

#### Do Black and Hispanic People Differ in Their PrEP Knowledge, Usage, and Interest?

Black participants (*M* = 2.12, *SD* = 0.87) were more familiar with PrEP compared to Hispanic participants (*M* = 2.36, *SD* = 0.86), *t*_(362)_ = −2.23, *p* = 0.03, *d* = 0.28. Among 275 Black participants, 44.7% had never heard of PrEP. Among 89 Hispanic participants, 60.7% had never heard of PrEP. Black participants (*M* = 1.96, *SD* = 0.35) were also more likely to currently be on PrEP compared to Hispanic participants (*M* = 2.04, *SD* = 0.30), *t*_(361)_ = −1.97, *p* = 0.05, *d* = 0.24. However, Black (*M* = 3.38, *SD* = 1.19) and Hispanic (*M* = 3.40, *SD* = 1.25) participants' interest in PrEP after learning of it did not significantly differ, *t*_(338)_ = −0.13, *p* = 0.90. See Table 3 for means and standard deviations for each predictor by race/ethnicity and Table 4 for frequencies for each predictor by race/ethnicity.

**Table 3:** Means and standard deviations for the predictors by race/ethnicity.

	**Black participants**			**Hispanic participants**		
**Predictor**	**M**	**SD**	***n***	**M**	**SD**	***n***
Knowledge of PrEP	2.12	0.87	275	2.36	0.86	89
Interest in PrEP	3.38	1.19	253	3.40	1.25	87
Usage of PrEP	1.96	0.35	274	2.04	0.30	89

**Table 4:** Frequency, interest, and usage of PrEP among black and hispanic participants.

	**Black participants (*n* = 275)**	**Hispanic participants (*n* = 89)**
**Before today have you heard of PrEP? (** ***n)***		
Yes	32.4% (89)	24.7% (22)
Yes, but I didn't know what it was for	22.9% (63)	14.6% (13)
No	44.7% (123)	60.7% (54)
**Are you interested in getting PrEP for yourself? (** ***n)***		
Definitely yes	29.8% (82)	9.0% (8)
Probably yes	20.4% (56)	12.4% (11)
Not sure	18.9% (52)	31.5% (28)
Probably not	18.9% (52)	20.2% (18)
Definitely not	18.9% (52)	25.7% (22)
Missing data	18.9% (52)	2.2% (2)
**Are you currently on PrEP? (** ***n)***		
Yes	8.0% (22)	2.2% (2)
No	87.3% (240)	91.0% (81)
No, but I have been in the past	4.4% (12)	6.7% (6)

#### Reported Preferences for Discussing PrEP in a Medical Setting

There was no significant difference between Black and Hispanic participants' interest in PrEP, *t*_(338)_ = −0.13, *p* = 0.90. 21.5% of Black participants and 21.4% of Hispanic participants were interested in PrEP. An additional 31.3% of Black participants and 31.5% of Hispanic participants were unsure of their interest in PrEP.

#### Reported Preferences for Asking About PrEP in a Medical Setting

The most endorsed strategy for improving patient comfort with discussing PrEP in a medical setting directly necessitates action on behalf of the provider. Nearly half (45.9%; *n* = 167) of the sample reported they would feel most comfortable if the medical provider was the one to bring up PrEP in the conversation, rather than the patient having to bring it up themselves. The other three most endorsed strategies revolved around increased education for the patient themselves: Nearly 30% (*n* = 109) would feel more comfortable discussing PrEP with their provider if they had more information about PrEP first, including easily accessible brochures in the lobby. Additionally, 31% (*n* = 113) of participants would feel more comfortable asking a doctor for PrEP if they already knew they qualified for it, and another 24.7% (*n* = 90) would feel more comfortable if they already knew their insurance covered the medication. See Table 5 for complete frequencies across individual race/ethnicities and in the total sample.

**Table 5:** Options for asking about PrEP more comfortable for the patient (select all).

	**Black participants (*n* = 275)**	**Hispanic participants (*n* = 89)**	**All participants (*N* = 364)**
**Similarity items (** ***n*** **)**			
Doctor same race as patient	13.1% (36)	4.5% (4)	11.0% (40)
Doctor same gender as patient	17.5% (48)	24.7% (22)	19.2% (70)
Doctor same religion as patient	6.9% (19)	3.4% (3)	6.0% (22)
Doctor similar age as patient	6.2% (17)	7.9% (7)	6.6% (24)
Doctor same sexuality as patient	7.3 % (20)	11.2% (10)	8.2% (30)
**Financial items (** ***n*** **)**			
If patient knows they qualified for PrEP	29.8% (82)	34.8% (31)	31.0% (113)
If patient knows insurance covered PrEP	20.4% (56)	38.2% (34)	24.7% (90)
If patient knows they can afford PrEP	18.9% (52)	31.5% (28)	22.0% (80)
**Provider items (** ***n*** **)**			
If the provider brought PrEP up first	45.8% (126)	46.1% (41)	45.9% (167)
If the patient knew more about PrEP	28.4% (78)	34.8% (31)	29.9% (109)
If there were brochures in the lobby	20.0% (55)	20.2% (18)	20.1% (73)
If the patient knew they qualified for PrEP	5.1% (14)	3.4% (3)	31.0% (113)
If it were a doctor never visited before	8.4% (23)	3.4% (3)	7.1% (26)
If it were a doctor they've seen a lot	23.6% (65)	28.1% (25)	24.7% (90)
**Location items (** ***n*** **)**			
If patient lived in a bigger city	5.5% (15)	4.5% (4)	5.2% (19)
If patient lived in a different state	4.7% (13)	2.2% (2)	4.1% (15)
**Privacy items (** ***n*** **)**			
If provider won't tell anyone	8.0% (22)	9.0% (8)	8.2% (30)
If provider won't judge patient	12.7% (35)	21.3% (19)	14.8% (54)
If no one else knows their prescription	7.6% (21)	7.9% (7)	7.7% (28)

## General Discussion

In the present study we examined familiarity, interest, and usage of PrEP among Black and Hispanic Indiana residents. Between 45 and 60% of participants had never heard of PrEP prior to our survey, and only between 9 and 12% of participants had taken PrEP before. Considering that Black and Hispanic people have an emphasized risk for HIV but continue to have the least familiarity with PrEP as an HIV preventative, our results add to the call for immediacy in addressing the spread of HIV beyond a strict focus on men who have sex with men.

Black participants were more familiar with PrEP relative to Hispanic participants and were more likely to have been on PrEP before, although rates of use in both groups were very low. This low uptake of PrEP is likely due to the general lack of knowledge of PrEP, as we found that nearly half of the sample was unfamiliar. Our results are in line with research that suggests that general awareness of PrEP is low (36, 37). A recent study suggested that level of PrEP knowledge has converged for Black and white Americans, while Hispanic/Latinx Americans remain behind in knowledge (38). This has been further demonstrated specifically for Hispanic heterosexual women, who are less likely to be aware of PrEP but are amenable to taking PrEP to help with HIV prevention (29). This may account for the familiarity disparity among Black and Hispanic participants. Importantly, after learning about PrEP from our survey, around 21% of both Black and Hispanic participants were interested in pursuing PrEP for themselves.

We identified actionable ways to help patients feel more comfortable in discussing PrEP with medical providers. Nearly 46% of Black and Hispanic participants felt most comfortable with PrEP if their medical provider discussed the medication with them by bringing it up in a medical appointment. Our results demonstrate that many people would be willing to discuss PrEP and consider it as an option for them, if only their provider would broach the subject. There is a critical need for education and direct discussion regarding the function and suitability of PrEP within patient-provider relationships. Indeed, recent research has suggested that additional education for medical providers can support more direct accessibility to PrEP usage (39).

Additionally, Black and Hispanic participants wanted more information to increase their comfort level with PrEP. They wanted to know, prior to bringing up PrEP with their doctor, if they qualified for the medication and if their insurance would cover the medication. Participants also wanted more written information regarding PrEP (i.e., brochures). This form of educational material is easy to access and conceal and allows for reading in privacy. Future interventionists may wish to create fact sheets or brochures to encourage PrEP uptake.

Some participants (*n* = 80) in our study reported being unsure if they wanted to use PrEP. Additional conversations with a trusted medical provider may help them to decide if this medication is well-suited for their needs. Research on barriers to PrEP have identified concerns around lack of knowledge regarding PrEP, including the perception that one may be a candidate for PrEP, the perceived cost of PrEP, and understanding the treatment regimen (25, 40). Medical providers play an important role in challenging these barriers, as they can provide information about eligibility for and insurance coverage of PrEP, and any potential options that could reduce the treatment burden (e.g., cabotegravir as PrEP, a once-every-8-weeks injection). Without this aid from the provider, many may be discouraged from asking for PrEP themselves.

Additionally, several participants (*n* = 54) indicated that they would feel more comfortable if they could trust that their provider was non-judgmental in discussing PrEP. A significant factor in predicting an individual's willingness to engage in HIV testing is the level of perceived stigma directed toward the individual [and associated psychological distress as a byproduct of this stigma; (41)]. This perceived stigma surrounding HIV has been extended specifically to PrEP usage, thus adding another layer of potential barriers surrounding the uptake and usage of PrEP (42). Stigma surrounding PrEP usage and HIV testing/treatment may manifest as prejudicial behavior and/or negative attitudes directed toward PrEP users, frequently rooted in misinformed stereotypes about those with HIV or those who would use PrEP. Medical practitioners who are educated about biases and prejudice surrounding those who have HIV, as well as those who utilize PrEP, can help establish a foundation of tolerance and acceptance, encouraging patients to feel more confident and comfortable in conversations about PrEP.

Of particular note, our sample was largely heterosexual. This is in direct contrast with the majority of HIV and PrEP research, which focuses specifically on MSM. However, this is a strength of the present research, because ~23% of new HIV diagnoses are due to heterosexual contact (11). Research examining HIV and gender further contextualizes this finding, as heterosexual contact accounts for 85% of HIV diagnoses among women nationwide (5). Relatedly, 51% of participants from this sample were Black women, a demographic that is heavily impacted by HIV diagnoses but rarely present in HIV literature and in medical spaces regarding HIV prevention techniques. In this way, this study provides important insights for how to increase PrEP uptake among Black women.

In terms of limitations, our sample included only 89 Hispanic participants. Although these participants were able to offer valuable, unique insight into PrEP contexts, future research should work to recruit larger, balanced samples of Hispanic, Black, and Black/Hispanic identifying individuals. The current study also only provided the survey questions in English, which may have not been the preferred language of some participants so future research should provide an option in Spanish for Hispanic individuals. Future studies would also benefit from using more robust scalar measurements of concepts; in the present research, we utilized single-item measures of PrEP use, familiarity, and interest to reduce participant time burdens. Validated scales will provide more reliable and comparative data.

## Conclusion

We investigated PrEP familiarity, prior usage, and interest in using PrEP in a sample of Black and Hispanic Indiana residents. Around half of the sample was unfamiliar with PrEP, and 21% of the sample reported interest in PrEP after learning. Interest was similar in Black and Hispanic participants, but familiarity with PrEP was lower for Hispanic participants. Medical providers directly addressing PrEP, identifying qualified patients, and providing reading material (i.e., brochures) are all potential avenues identified by our participants for increasing their comfort with broaching the topic of PrEP.

## Data Availability Statement

The raw data supporting the conclusions of this article will be made available by the authors, without undue reservation.

## Ethics Statement

The studies involving human participants were reviewed and approved by the IRB at Indiana University. The patients/participants provided their written informed consent to participate in this study.

## Author Contributions

JC, OA, MB-B, BW, AG, and GC: conceptualization, investigation, and writing—review and editing. GC and BW: methodology. JC: software, formal analysis, and visualization. JC, MB-B, and AG: validation. JC and AG: writing—original draft preparation. All authors have read and agreed to the published version of the manuscript.

## Funding

This study was funded by a grant from the Indiana Minority Health Association.

## Conflict of Interest

The authors declare that the research was conducted in the absence of any commercial or financial relationships that could be construed as a potential conflict of interest.

## Publisher's Note

All claims expressed in this article are solely those of the authors and do not necessarily represent those of their affiliated organizations, or those of the publisher, the editors and the reviewers. Any product that may be evaluated in this article, or claim that may be made by its manufacturer, is not guaranteed or endorsed by the publisher.
